# NeuroHeal Reduces Muscle Atrophy and Modulates Associated Autophagy

**DOI:** 10.3390/cells9071575

**Published:** 2020-06-28

**Authors:** Sara Marmolejo-Martínez-Artesero, David Romeo-Guitart, Laura Mañas-García, Esther Barreiro, Caty Casas

**Affiliations:** 1 Institut de Neurociències (INc) and Department of Cell Biology, Physiology and Immunology, Universitat Autònoma de Barcelona (UAB), Bellaterra, 08193 Barcelona, Spain; Sara.Marmolejo@uab.cat; 2 Pulmonology Department-Muscle Wasting and Cachexia in Chronic Respiratory Diseases and Lung Cancer Research Group, IMIM-Hospital del Mar, Parc de Salut Mar, Health and Experimental Sciences Department (CEXS), Universitat Pompeu Fabra (UPF), Barcelona Biomedical Research Park (PRBB), 08003 Barcelona, Spain; lauramgarcia9@gmail.com (L.M.-G.); ebarreiro@imim.es (E.B.); 3 Centro de Investigación en Red de Enfermedades Respiratorias (CIBERES), Instituto de Salud Carlos III (ISCIII), 08003 Barcelona, Spain

**Keywords:** NeuroHeal, skeletal muscle atrophy, sirtuin 1, autophagy, proteasome

## Abstract

Muscle wasting is an unmet medical need which leads to a reduction of myofiber diameter and a negative impact on the functional performance of daily activities. We previously found that a new neuroprotective drug called NeuroHeal reduced muscle atrophy produced by transient denervation. Aiming to decipher whether NeuroHeal has a direct role in muscle biology, we used herein different models of muscle atrophy: one caused by chronic denervation, another caused by hindlimb immobilization, and lastly, an in vitro model of myotube atrophy with Tumor Necrosis Factor-α (TNFα). In all these models, we observed that NeuroHeal reduced muscle atrophy and that SIRT1 activation seems to be required for that. The treatment downregulated some critical markers of protein degradation: Muscle Ring Finger 1 (MuRF1), K48 poly-Ub chains, and p62/SQSTM1. Moreover, it seems to restore the autophagy flux associated with denervation. Hence, we envisage a prospective use of NeuroHeal at clinics for different myopathies.

## 1. Introduction

Skeletal muscle atrophy or muscle wasting can occur due to several causes, from short periods of muscle disuse due to primary degenerative processes within the skeletal muscle fibers or to secondary neurogenic atrophy and inflammation or spontaneously during aging [1]. The hallmark histopathological feature of skeletal muscle atrophy is the loss or reduction of myofiber diameter. Loss of muscle mass results in reduced muscle function, and this can have a direct negative impact on functional performance, such as the ability to carry out activities of daily living. Muscle wasting has also been associated with poor prognosis and mortality [2]. Medications that treat or prevent muscle atrophy remain mostly unavailable, with exercise and/or physical therapy being the only remaining options.

Optimal muscle state depends on the balance between protein synthesis and protein degradation. Muscle atrophy is initially provoked by an excessive protein breakdown, followed by a reduction in protein synthesis [3]. Several reports describe that increasing protein synthesis could be beneficial and sufficient to block muscle weight loss. Moreover, it is widely described that an overactivation of proteasome or autophagic-degradative pathways hampers muscle tissue. Although this is true regarding proteasome-mediated protein degradation, recent studies suggest that macroautophagy, hereafter autophagy, could have a muscle-protective effect instead. Specifically, the blockade of autophagy has been related to exacerbated atrophy, indicating some beneficial effects of this self-protective mechanism [4].

We have recently discovered a novel neuroprotective drug, NeuroHeal, for peripheral nerve injury (PNI). The drug, discovered using artificial intelligence, is based on the combination of two approved drugs, acamprosate and ribavirin, which facilitate its readiness for clinical use [5]. Using a model of severe PNI with delay reimplantation of the damaged nerve roots, we observed that the treatment with NeuroHeal enhanced nerve regeneration and reduced the associated muscle atrophy [6]. Considering the neuroprotective effect of NeuroHeal, the expected reduction of muscle atrophy might be correlated. In order to know whether NeuroHeal might have a direct effect on muscle biology, we set up a series of experiments with specific models of muscle atrophy in the present report. The findings obtained opened exciting possibilities for its use at clinics for different types of myopathies.

## 2. Materials and Methods

### 2.1. Denervation Model

All the procedures involving animals were approved by the ethics committee of the Universitat Autònoma de Barcelona and Generalitat de Catalunya and followed the European Community Council Directive 2010/63/EU. Female Sprague–Dawley rats weighted at 220–250 g at 10 weeks old were purchased from Harlan Laboratories (Indianapolis, IN, USA). For surgical intervention, animals were under anesthesia by intraperitoneal injection of 90 mg/kg ketamine (Ketaset) and 10 mg/kg xylazine (Rompun) [6]. Upon exposure of the sciatic nerve freed of connections, a 2 cm of nerve section was transected above the sciatic nerve trifurcation. The wounds were sutured by planes and disinfected with povidone iodine, and the animals were allowed to recover in a warm environment.

### 2.2. Hindlimb Immobilization Model

All animal experiments were conducted in the animal facilities at Barcelona Biomedical Research Park (PRBB). This study was designed in accordance with the ethical standards on animal experimentation (EU 2010/63 CEE, Real Decreto 53/2013 BOE 34, Madrid, Spain). Ethical approval was obtained by the animal research committee (Animal welfare department in Catalonia, Spain, EBP-13-1485, 2015/04/20). Female C57BL/6J mice (10 weeks old and weight approximately 20 g) were obtained from Harlan Interfauna Ibérica SL (Barcelona, Spain). Mice were exposed to unilateral hindlimb immobilization as previously described to reproduce a model of disuse-induced muscle atrophy [7,8,9]. Briefly, the left hindlimb was shaved with clippers and was enveloped using surgical tape. The hindlimb was introduced in a 1.5 mL microcentrifuge tube with a cover and the bottom lids were removed, while maintaining the foot in a plantar-flexed position to induce the maximal atrophy of the target limb muscle. As the weight of the tube was approximately 0.6 g, it did not interfere with the usual mobility of the mice. The left hindlimb of the mice was immobilized for 7 consecutive days, with the time at which the splint was removed to let the animals move freely in their cages to evaluate muscle recovery being 7 days later.

### 2.3. Drug Treatment

The NeuroHeal mixture is composed of acamprosate and ribavirin. For the rat treatment, we ground acamprosate (Merck, Darmstadt, Germany) and ribavirin (Normon, Madrid, Spain) pills into fine powders and added both to drinking water at final concentrations of 2.2 mM and 1 mM, respectively. The groups treated with NeuroHeal and 5 mM nicotinamide (NAM) (Sigma-Aldrich, Saint Louis, MO, USA) were given water-containing drugs from the day of the surgery. Fresh drug solutions were made every 3 days. For the in vivo experiments with mice, we ground acamprosate (Merck, Darmstadt, Germany) and ribavirin (Normon, Madrid, Spain) pills into fine powder and administrated intraperitoneally daily 40 mg/kg and 26 mg/kg, respectively.

### 2.4. Grip Test

In all mice, limb strength was determined on day 0, day 7, and day 14, using a specific grip strength meter for rodents (Bioseb, Vitrolles Cedex, France) following previously published methodologies [10,11,12,13]. In all mice, the four limbs equally contributed to the evaluation of grip strength. In all the animals, limb strength gain was calculated as the percentage of measurements performed at the end of the study period with respect to the same measurements obtained at baseline (grip strength at the end of the study-period grip strength on day 0)/grip strength on day 0 × 100).

### 2.5. Histology

Rats were euthanized with Dolethal (Veotquinol, Madrid, Spain), and mice were euthanized with 0.1 mL sodium pentobarbital (60 mg/kg, intraperitoneally). In all cases, the pedal and blink reflexes were evaluated in order to verify total anaesthetic depth. Rats were perfused with a transcardial infusion of a saline solution containing heparin (10 U/mL) followed by 4% paraformaldehyde in 0.1 M Phosphate Buffered Saline (PBS). Gastrocnemius (GA) muscles (ipsi- and contralateral) were obtained from all the animals after perfusion and were weighted. From mice, the muscle samples were weighted and directly fixed in 4% paraformaldehyde solution. All samples were embedded thereafter into a 30% sucrose solution for cryopreservation at 4 °C until needed.

We transversally cut the GA in the middle, placed them in Tissue-tek for serial cutting (10 µm sections) in a cryotome (Leica, Heidelberg, Germany) to obtain 20 slides with 5 cuts, and stored them at −20 °C until analysis. The distance between the sections of each slide was 100 µm, covering a total of 500 µm of each muscle. Histology and immunohistochemistry of the sections to be compared between them were processed together the same day within the same slide, and image analyses for all groups were also performed the same day. Transversal GA muscle sections were stained with Hematoxylin and Eosin (H&E). Briefly, nuclei were stained with Harris hematoxylin for 6 min followed by differentiation with acid solution of 0.01% HCl in ethanol. Cytoplasm was stained with eosin for 1 min. Sections were dehydrated by graded ethanol (50%, 70%, 96%, and 100% and xylene twice, 5 min each solution) and mounted with DPX mounting medium. For mice tissue analysis, we randomly took images under light microscopy at 20× (Nikon Eclipse Ni-E, Nikon, Tokyo, Japan) equipped with a digital camera (Nikon DS-RiE, Nikon, Tokyo, Japan) and Nikon NIS-Element BR software (version 5.11.03, Nikon, Tokyo, Japan). For rat tissue analysis, we randomly took images under light microscopy at 20× (Olympus BX51, Olympus, Hamburg, Germany) equipped with a digital camera (Olympus DP50, Olympus, Hamburg, Germany) and CellSens Digital Imaging software (version 1.9, Olympus, Hamburg, Germany). For cross-sectional area (CSA) determination, at least 100 muscle fibers from three different images per muscle were measured. The mean and area distribution for each animal was analyzed (*n* = 4 per group) using Image J software (version 1.46; National Institutes of Health; available at http://rsb.info.nih.gov/ij/), and the minimum diameter and the circular area were calculated based on a ratio of calibrated pixels to actual size (mm).

For immunofluorescence labeling, the slices were pretreated with 10 mg/mL NaBH_4_ to reduce autofluorescence for 80 min at 4 °C. After washing with standard Phosphate Buffered Saline (PBS), the tissue was incubated in a blocking solution (0.3% Triton-X-100 and 10% fetal bovine serum in PBS) for 1 h at room temperature. Samples were then incubated overnight at 4 °C with primary antibody rabbit anti-acetyl H4K16 (H4K16Ac; 1:1000, Millipore, Burlington, MA, USA) and mouse anti-laminin (1:100, DSHB, Iowa City, IA, USA) After several washes with 0.3% Triton-X-100 in PBS, we added Alexa Fluor 488 against the primary antibody (1:200; Jackson Immunoresearch, Cambridge, UK) and incubated for 1 h at room temperature. Counterstaining was performed with DAPI (Sigma-Aldrich), and mounting was performed with Mowiol mounting medium (Southern Biotech, Birmingham, UK). Images of the muscle samples from different groups were taken under the same exposure time, sensibility, and resolution for each marker analyzed with the aid of a digital camera (Nikon D5-Ri2) attached to the microscope (Nikon ECLIPSE Ni) and NIS-Elements BR software (version 5.11.03). Co-labeled fibers were determined as positive using a pseudocolor display by Image J software (version 1.46; National Institutes of Health).

### 2.6. Immunoblotting

For immunoblotting, denervated rats were euthanized with Dolethal (60 mg/kg, intraperitoneally) at 7 days post-injury (dpi), and the right GA muscle was obtained, snap-frozen in liquid nitrogen, and stored at −80 °C. GA half-quarter was added to the lysis buffer (50 mM Tris, pH 6.8, 2 mM EDTA, 0.5% Triton-X-100, and a cocktail of proteases (Sigma-Aldrich) and phosphatase inhibitors (Roche, Basel, Switzerland)), homogenized with a Pellet pestle (Sigma-Aldrich) on ice, and sonicated with an Ultrasonic homogenizer (Model 3000, Biologics Inc., Manassas, VA, USA). Samples were centrifuged for 10 min at 13,000 *g* at 4 °C, and protein in supernatants were quantified by BCA assay (Pierce Chemical Co., Dallas, TX, USA). Proteins (20 µg/well) were resolved by SDS-PAGE and transferred to a PVDF membrane in a BioRad cuvette system in 25 mM Tris, pH 8.4, 192 mM glycine, and 20% (*v*/*v*) methanol. We blocked the membrane with 6% milk solution in 0.1% Tween-TBS (TBS-T) for 1 h at room temperature and incubated it overnight with primary antibodies: rabbit anti-phospho-Ulk1 (Ser 555) (pUlk1; 1:1000; Millipore), mouse anti-Atg5 (1:1000; Nanotools, Teningen, Germany), rabbit anti-LC3 (1:1000; Abcam, Cambridge, UK), mouse anti-p62 (1:100; BD Transduction Laboratories New Jersey, USA), rabbit anti-phospho-AKT (Ser 473) (pAKT; 1:1000; Cell Signaling Technology, Danvers, MA, USA), rabbit anti-AKT (1:1000; Cell Signaling Technology), rabbit anti-phospho-S6 kinase (Thr389) (pP70S6k; 1:1000, Antibodies Online, USA), mouse anti-Muscle Ring Finger 1 (MuRF1) (1:100, Santa Cruz Biotechnology, Dallas, TX, USA), mouse anti-Atrogin-1 (1:100 Santa Cruz Biotechnology), mouse anti-Ub-k48 (1:100), rabbit anti-s5α (1:1000), and mouse anti-GAPDH (1:5000; Sigma-Aldrich). After washing in TBS-T, the membrane was incubated with an appropriate secondary antibody conjugated with horseradish peroxidase (1:5000, Vector Laboratories, Burlingame, CA, USA) for 1 h. The membrane was visualized using a chemoluminiscent method (ECL Clarity Kit, Bio-Rad Laboratories, Hércules, CA, USA), and the images were captured and quantified with Image Lab Software (Bio-Rad Laboratories).

### 2.7. In Vitro Experiments

The C2C12 myoblast cell line was grown in medium composed by modified Eagle’s medium high-glucose (DMEM, Life Technologies, Carlsbad, CA, USA) supplemented with 10% fetal bovine serum (Sigma-Aldrich) and 1% penicillin/streptomycin solution (Sigma-Aldrich). Cells were kept in a humidified incubator at 37 °C under 5% CO_2_. To initiate the experiments, we seeded the cells at a density of 8.5 × 10^3^ cell/mL, and after 24 h of culture, the medium was changed to a differentiation medium (DMEM supplemented with 2% horse serum (Sigma-Aldrich) with 1% penicillin/streptomycin solution), which was changed every 2 days. After 6 days of in vitro culture, these cells differentiated and fused to form myotubes. At this time, we added different drugs (acamprosate, ribavirin, Ex-527 (Sigma-Aldrich), and Tumor Necrosis Factor-α (TNFα, Sigma-Aldrich) at 55 μM, 1 μM, 10 μM, and 100 ng/mL, respectively) to the cells for 24 h. Thereafter, cells were fixed using 4% tamponed formaldehyde for 30 min and then washed out with TBS for cytology observations. For immunoblotting, we added RIPA buffer (50 mM tris-HCl, 1% triton-X-100, 0.5% Na-deaxycholate, 0.1% SDS, 150 mM NaCl, and 2 mM EDTA) with inhibitor phosphatases 20× and inhibitor proteases 100× buffer to the cultured cells (*n* = 4–5). For the fiber diameter evaluation, we randomly took images under light microscopy at 20× (Olympus CKX41, model U-LH50HG) equipped with a digital camera (Olymus U-TV1x-2) and an Olympus DP20-5 device from three wells for each condition for at least 100 myotubes for each condition. The area distribution for each condition was analyzed (*n* = 3 per group) using Image J software (version 1.46; National Institutes of Health), was calculated using the circle area equation and using the length between two points from the myotubes width below the nucleus using the “straight” tool based on a ratio of calibrated pixels to actual size (mm).

### 2.8. Statistics

The normality and homoscedasticity of the data were studied for each parameter to apply the correct statistical method (parametric or nonparametric) using the Shapiro–Wilk test. For all the data, we performed an unpaired Student’s t-test to compare two groups, one-way analysis of variance (ANOVA) to compare three or more groups, and two-way analysis of variance (ANOVA) to compare grouped data followed by Turkeys’ multiple comparison test. Data are presented as means ± standard error of the mean (SEM). Statistical analyses were conducted using GraphPad Prism 8 software (San Diego, CA, USA). The myofiber and myotube distributions did not present normality, and we performed Kuskal–Wallis test with Benjamin, Krieger and Yekutieti post hoc. Data are presented as mean, and statistical analyses were conducted using GraphPad Prism 8 software. Differences were assumed to be significant for *p* ≤ 0.05.

## 3. Results

### 3.1. NeuroHeal Reduces Atrophy-Related Histological Parameters

**Figure 1:**
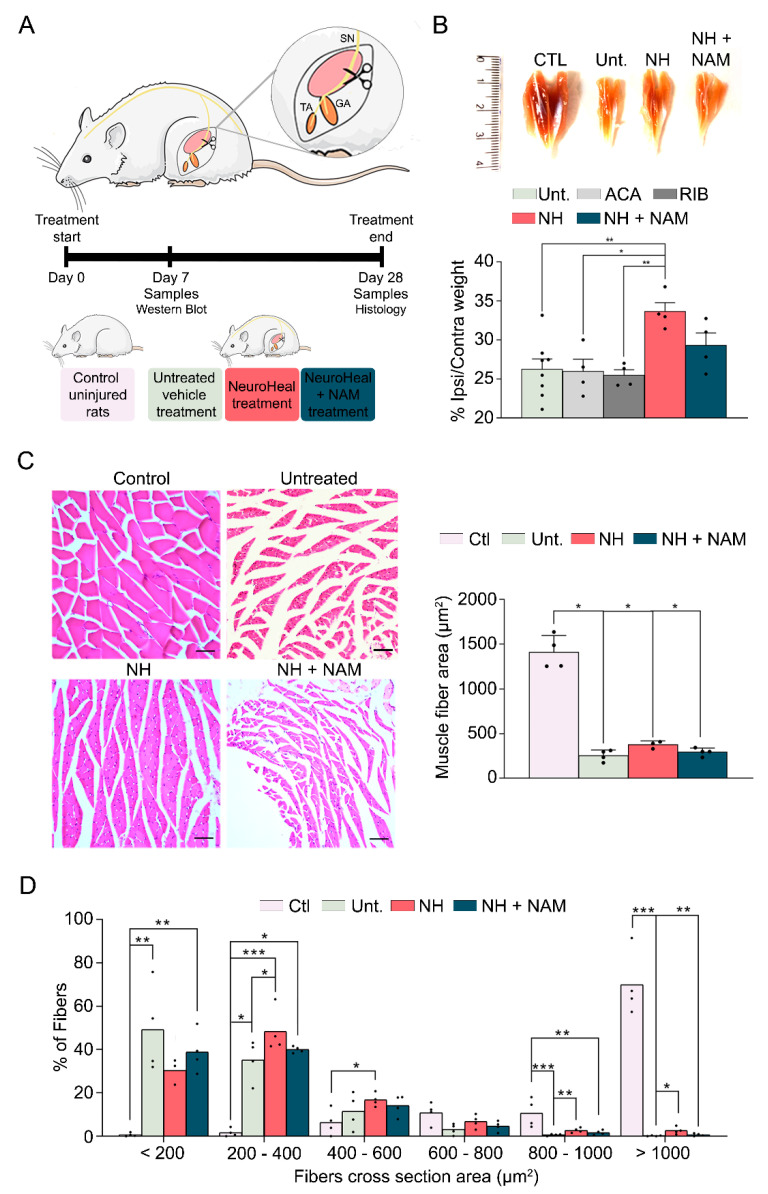
NeuroHeal treatment reduces muscle atrophy by denervation. (**A**) Schematic workflow and experimental groups and (**B**) representative photographs of the gastrocnemius (GA) muscles removed from different experimental groups: control (CTL), injured untreated (Unt.), injured treated with NeuroHeal (NH), and injured treated with NH plus nicotinamide (NAM). Bar graph of the relative average weight of the ipsilateral muscle with respect to the contralateral one from different groups including those animals treated with acamprosate (ACA) or ribavirin (RIB) alone at 28 days post-injury (dpi) (*n* = 4; one-way ANOVA, * *p* < 0.05) is shown. (**C**) Left, representative microphotographs of cross GA muscle sections with Hematoxylin and Eosin (H&E) staining at 28 dpi (scale bar = 100 µm) and, right, representation of cross-sectional area (µm^2^) mean of all fibers in the GA muscle (*n* = 4; one-way ANOVA * *p* < 0.0001). (**D**) Histogram of the cross-sectional area (µm^2^) distribution of fibers in the GA muscle of different groups (*n* = 4; Kruskal–Wallis, Benjamin, Krieger, and Yekutieti post hoc * *p* < 0.05).

First, we attempted to decipher the adverse effects of NeuroHeal on healthy animals, so we treated control rats with it for 14 days and analyzed muscle weight and cross-sectional area (CSA). Healthy muscles treated with NeuroHeal did not show changes in muscle weight or fiber size, indicating that NeuroHeal does not have a detrimental impact on healthy muscles (Figure S1). Then, we analyzed the effect of NeuroHeal on muscle atrophy using a model of chronic denervation by sciatic nerve transection. The experimental design consists of 4 groups of animals, as shown in Figure 1A: control-uninjured animals, injured animals treated with vehicle, injured animals treated with NeuroHeal, and injured animals treated with NeuroHeal plus nicotinamide (NAM). Some animals per group were sacrificed at 7 days post-injury (dpi) for western blot analyses, and others were sacrificed at terminus 28 dpi. The last group was included using NAM, which inhibits SIRT1, in order to know whether this protein is involved in any effect promoted by NeuroHeal. Indeed, we verified that the abundance of one of the substrates deacetylated by SIRT1, the histone H4 at lysine 16 (H4K16ac), was modulated in the injured muscles by the treatment (Figure S2). Denervation did not significantly affect the number of H4K16ac-positive nuclei per area at the GA, whereas this number significantly decreased in the group treated with NeuroHeal, as expected by an inducer of SIRT1 activity. In agreement, co-treatment with NAM abolished the effect of NeuroHeal (Figure S2).

Muscle wasting of 26% ± 1.31%, depicted by a reduced muscle mass, was observed in the ipsilateral GA compared to the contralateral side at 28 dpi (Figure 1B). A similar reduction was observed in the groups of animals treated with acamprosate and ribavirin per separate, the compounds of NeuroHeal (acamprosate (ACA) 26% ± 1.54%; ribavirin (RIB) 25.47% ± 0.71%). In contrast, the NeuroHeal group presented significantly reduced muscle weight loss (33.63% ± 1.14%) compared with untreated animals. This attenuation in muscle wasting was not achieved when adding NAM to the treatment (29.3% ± 1.6%), suggesting that SIRT1 activity was involved in the action of NeuroHeal in muscle.

The skeletal muscle atrophy was also apparent at the single fiber level measured by myofiber CSA in all the denervated groups compared to the control group (Figure 1C). However, the distribution of fibers depending on their size varied among groups with a clear leftward shift due to denervation (Figure 1D). The untreated group showed a clustering of smaller fibers being mostly (49.23% ± 10.16%) below 200 µm^2^. The shift was less pronounced in the group of animals treated with NeuroHeal which presented the most abundant fibers in the range between 200 and 400 µm^2^ (48.31% ± 5.06%). Moreover, the prevalence of the bigger fibers with >800 µm^2^ CSA were significantly more abundant in the NeuroHeal group with respect to the untreated one. All these effects promoted by NeuroHeal treatment were not observed in the NeuroHeal plus NAM group which had a superior percentage of small fibers, <200 µm^2^, and less of the gross fibers, >1000 µm^2^, than the NeuroHeal group. Altogether, these results suggested that NeuroHeal treatment reduced or prevented myotrophy in a model of muscle chronic denervation.

### 3.2. NeuroHeal Reduces Muscle Atrophy in a Model of Mechanical Unloading

**Figure 2:**
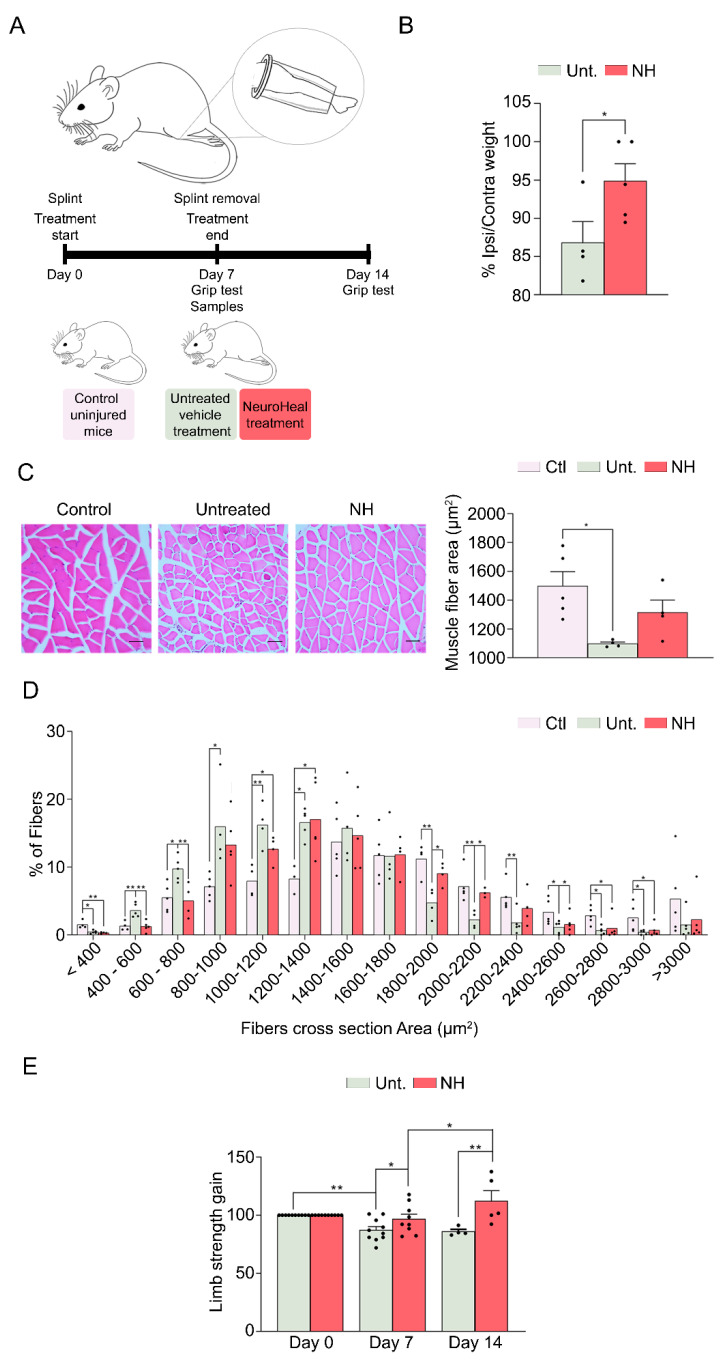
NeuroHeal treatment reduces muscle atrophy by disuse. (**A**) Schematic workflow and experimental groups and (**B**) a bar graph of the gastrocnemius (GA) muscle weight ratio between ipsilateral and contralateral sites at day 7 (*n* = 3–4; t-test, * *p* < 0.05). (**C**) Left, representative microphotographs of cross GA muscle sections with H&E staining at day 7 (scale bar = 100 µm) and, right, representation of cross-sectional area (µm^2^) mean of all fibers in the GA muscle (*n* = 5; one-way ANOVA * *p* < 0.05). (**D**) Histogram of the distribution of the cross-sectional area (µm^2^) of GA fibers (*n* = 3–4; Benjamin, Krieger, and Yekutieti post hoc * *p* < 0.05). (**E**) Bar graph of the grip strength of the immobilized hindlimb from NeuroHeal treated (NH) and untreated groups (Unt.) obtained at day 0, day 7, and day 14 (*n* = 5–10; one-way ANOVA, * *p* < 0.05).

We wanted to discern whether NeuroHeal was attenuating muscle atrophy only in cases with neurogenic origin or whether it may act similarly in those of non-neurogenic origin. Therefore, we used a different model to cause muscle atrophy based on short-term (one-week) hindlimb immobilization with the workflow experimentation schedule depicted in Figure 2A. After one week of recovery time, muscle wasting was evident with a reduction of up 86.82% ± 2.77% in the GA weight ratio of the ipsilateral muscle compared to the non-immobilized contralateral side. In contrast, animals treated with NeuroHeal maintained muscle weight to 94.88% ± 2.25%, significantly higher than untreated animals (Figure 2B). Accordingly, disuse caused a drastic reduction of the mean myofiber CSA in the immobilized and untreated with respect to the non-immobilized muscles although this was not so evident in the group treated with NeuroHeal (Figure 2C). Detailed examination of the distribution of the CSA showed a leftward shift in the fiber diameter distribution, with more prevalence of the smaller fibers, from 400 to 1200 µm^2^ (CTL 23.33%; Unt. 45.92%; NH 32.47%), and less abundance of those bigger than 1800 µm^2^. This shift was significantly less pronounced in the animals treated with NeuroHeal, with more abundance of the bigger sized fibers, >1800 µm^2^ (24.63%), than in the untreated animals (12.33%) (Figure 2D).

Finally, we performed a functional test using the grip test to evaluate the limb strength (Figure 2E). After 1 week of immobilization, the untreated group showed a decrease in strength in the four limbs, which was the worst after 1 week of recovery (Figure 2E; day 7, 87.15% ± 3.08%; day 14, 85.92% ± 1.84%). In contrast, the group treated with NeuroHeal showed a significantly higher strength in the immobilization week and even superior strength after the recovery week (Figure 2E; day 7, 96.7% ± 4.27%; day 14, 112.3% ± 8.95%).

### 3.3. NeuroHeal Preserved Fiber Diameter in Atrophied Myotubes Cells In Vitro

**Figure 3:**
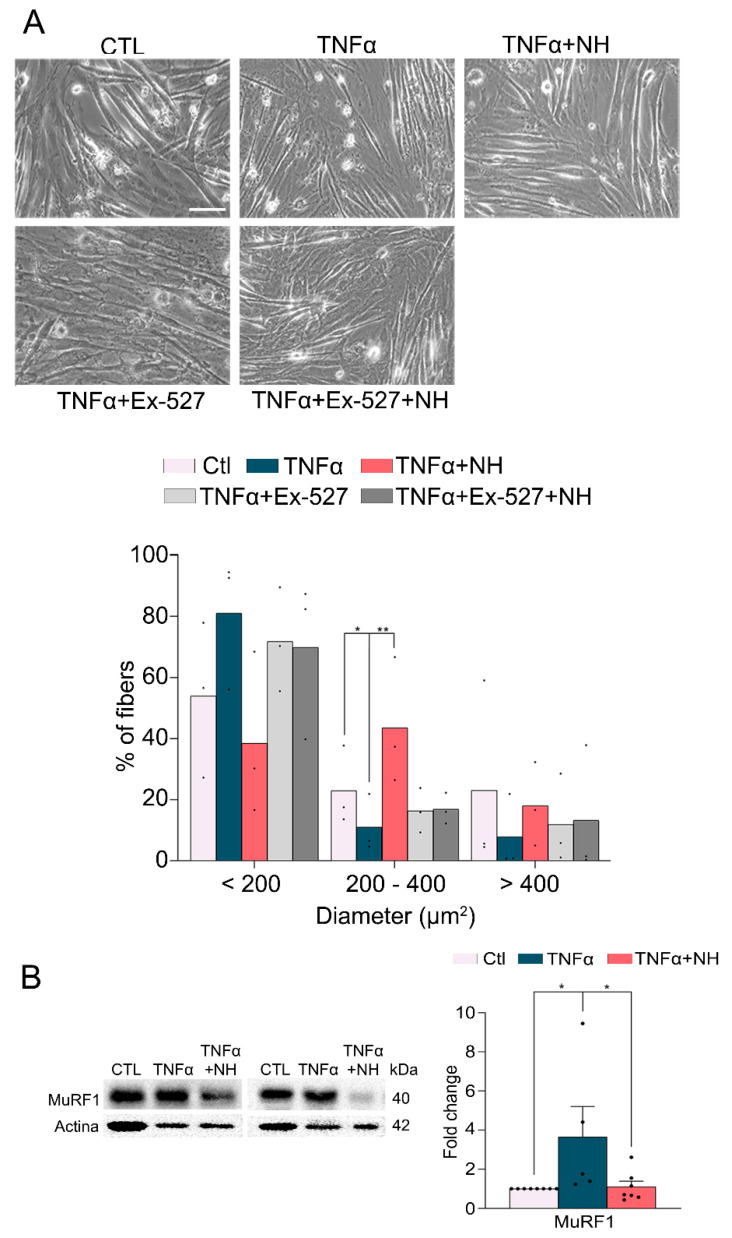
NeuroHeal reduces fiber atrophy produced by Tumor Necrosis Factor-α (TNFα) in vitro. (**A**) Representative microphotographs of myotubes derived from a C2C12 myoblast cell line culture at day 7 and treated during the last 24 h with different components (control (CTL), NeuroHeal (NH), and Ex-527 as a specific SIRT1 inhibitor (scale bar = 50 µm)). Histogram of the distribution of myotubes diameter (*n* = 3–6; Kruskal–Wallis, Benjamin, Krieger, and Yekutieti post hoc, * *p* < 0.05) and (**B**) a Western blot and the corresponding bar graph showing the analyses of Muscle Ring Finger 1 (MuRF1) protein levels in the different experimental groups at day 7 (*n* = 3; one-way ANOVA, * *p* < 0.05).

To confirm that the action of NeuroHeal was produced on the muscular cells independently of a nervous compound, we used a model of myoblast C2C12 cells which form myotubes after 7 days of culture in differentiation medium in vitro [14]. We treated the differentiated myotubes with 10 µg/mL TNFα for 24 h, with or without NeuroHeal or Ex-527, a specific SIRT1 inhibitor. Analyzing the diameter of the formed myotubes, we observed that TNFα caused a shift in the diameter of fibers, increasing the amount of smaller fibers, those <200 µm^2^ (81.05% ± 12.49% vs. 53.93% ± 14.68% in the control, non-atrophied cells), and diminishing the amount of the bigger ones, >400 µm^2^ (7.86% ± 7.04% vs. 23.1% ± 18% in the control, non-atrophied cells) (Figure 3A). In contrast, the treatment addition of NeuroHeal for the same time as TNFα sustained most of the fibers in its median size (between 200 and 400 µm^2^) (43.5% ± 12.01%) or bigger sizes (18.02% ± 7.9%). The addition of the SIRT1 inhibitor abolished these effects promoted by NeuroHeal. Thus, in agreement with what was observed in vivo, NeuroHeal reduces atrophy-induced in muscle cells. We also observed that NeuroHeal treatment reduced the levels of the Muscle Ring Finger 1 (MuRF1), an E3 ligase used as a marker of atrophy in muscle (Figure 3B).

### 3.4. NeuroHeal Reduced Proteasome Markers

**Figure 4:**
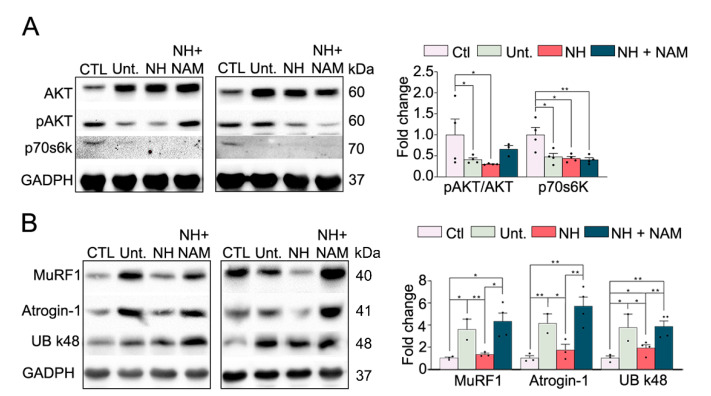
Ubiquitin-proteasomal degradation is modulated by NeuroHeal in the denervated muscle model. (**A**) Western blots and associated bar graphs showing the analyses of phosphorylated AKT, AKT, and pP70s6k protein levels in different experimental groups at 7 dpi (control (CTL), injured untreated (Unt.), injured treated with NeuroHeal (NH), and injured treated with NH plus nicotinamide (NAM)) (*n* = 3–4; one-way ANOVA, * *p* < 0.05) and (**B**) Western blots and associated bar graphs showing the analyses of Atrogin-1, MuRF1, and K-48 polyubiquitin chain protein levels in different experimental groups at 7 dpi (*n* = 3–4; t-test, * *p* < 0.05).

Since muscle atrophy is the result of a protein degradation rate which exceeds protein synthesis [15], we examined how NeuroHeal affected the balance between catabolism and anabolism. PI3K/Akt signaling stimulates the rate of protein synthesis via p70S6Kinase and p90 ribosomal S6 kinase and negatively regulates protein degradation, predominantly by its inhibiting effect on proteasomal and lysosomal protein degradation [1]. We observed that the ratio of phospho-AKT (Ser 473) isoform was reduced due to denervation at 7 dpi (Figure 4A). In agreement with a reduced activity of AKT, the levels of p70s6k were also reduced. The presence of NeuroHeal, with or without NAM, did not significantly change these effects.

On the other side, the prime system for muscle protein degradation is the ubiquitin-proteasome system [1]. During protein degradation, contractile proteins are ubiquitinated by the consecutive actions of the E1, E2, and E3 enzymes which can then be recognized and subsequently degraded by proteasomes. The gene expression as well as their function in muscle atrophy of two E3 ligases, MuRF1 and muscle atrophy F-box (MAFbx, also known as Atrogin-1), have been extensively examined. Both E3 ligases are particularly involved in the degradation of contractile proteins. During several atrophic conditions, MuRF1 and Atrogin-1 expression levels are increased [16]. Accordingly, we observed that MuRF1 and Atrogin-1 levels were raised in the injured untreated animals (Figure 4B). In contrast, the levels of both proteins were diminished in the NeuroHeal group which was abolished in the group with NAM. These results are in agreement with the reduced atrophy observed in denervated muscles.

In light of these differences, we further investigate the proteasome function by analyzing the K48-polyubiquitin chains which are the most abundant linkage in cells and are thought to be the major signal for proteasome-mediated degradation [17]. There was an increase in K-48 polyubiquitin chains in the lesioned untreated animals which was modified by NeuroHeal administration. In contrast, the addition of NAM considerably increased the levels of this factor (Figure 4B). At the same time, untreated injured animals showed an increase in the proteasome subunit 5α (s5α) [18], while NeuroHeal-treated animals presented normalized levels of it (Figure S3). These effects were reverted when the animals were co-treated with NeuroHeal and NAM.

Altogether, these results suggested that NeuroHeal did not substantially affect the protein synthesis, but NeuroHeal modulates the proteasome, which might lead to reduced atrophy.

### 3.5. NeuroHeal Modulates Atrophy Associated Autophagy

**Figure 5:**
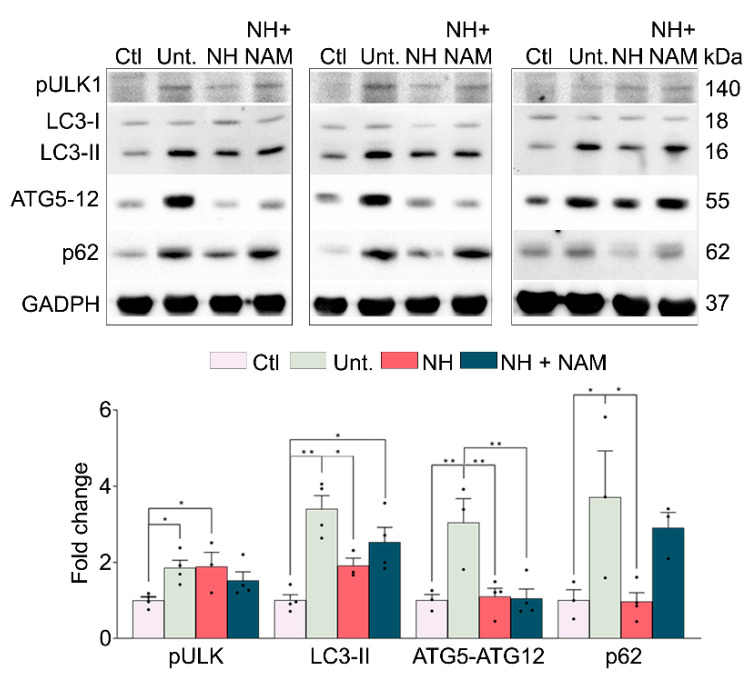
Autophagy flux is modulated by NeuroHeal in the denervated muscle model. Western blots and the associated bar graphs showing the analyses of phosphorylated ULK1, LC3-II, the ATG5-ATG12 complex, p62, and protein levels in different experimental groups at 7 dpi (control (CTL), injured untreated (Unt.), injured treated with NeuroHeal (NH), and injured treated with NH plus nicotinamide (NAM)) (*n* = 3–4; one-way ANOVA, * *p* < 0.05).

Autophagy is another key mechanism for muscle protein degradation [19]. Autophagy concerns the engulfment of cellular particles into autophagosomes, which subsequently fuse with lysosomes to be degraded in the acid intralysosomal environment. In cells undergoing autophagy, phagophore formation initiates after the Unc-51-like autophagy-activating kinase 1 (ULK1) activation, and its elongation is regulated by two ubiquitin-like reactions: the first leading to the formation of the complex ATG12-ATG5-ATG16L1 and the second involving the conjugation of the microtubule-associated protein light chain 3 (MAP-LC3/ATG8/LC3) to phosphatidylethanolamine at the autophagosome membrane to form autophagosome-associated LC3-II. Once the autophagosome is formed, it acquires the ability to bind autophagic substrates and/or proteins which mediate cargo selectivity (including sequestosome 1 (p62/SQSTM1)). We recently observed that NeuroHeal neuroprotects neonatal axotomized motoneurons and promotes autophagy [20]. Thus, we aimed to analyze whether this process was also involved in the observed reduction of muscle atrophy by NeuroHeal. All the protein levels of the markers analyzed, pULK1 (phosphorylation at Ser 555), LC3-II, ATG5-ATG12 conjugate, and p62/SQSTM1, were increased due to denervation as expected [21] (Figure 5). The activation of pULK1 was sustained in the NeuroHeal group; however, the levels of LC3-II were reduced and those of ATG5-ATG12 conjugate and p62/SQSTM1 were normalized. The addition of NAM did not promote differences in these markers with respect to NeuroHeal treatment alone except for a striking increase in p62/SQSTM1 levels (Figure 5). Altogether, these results suggested that denervation caused an activation of autophagy characterized by LC3II and p62/SQSTM1 levels which may suggest autophagy flux blockade, whereas the reduction promoted by NeuroHeal pointed to a better resolution of this process. SIRT1 activation by NeuroHeal may be involved particularly in the diminution of p62/SQSTM1.

## 4. Discussion

Herein, we have demonstrated that NeuroHeal reduces neurogenic and non-neurogenic myopathy using diverse models: a model of chronic denervation, a disuse model, and an in vitro model without the influence of neuronal components. Analyzing the anabolic/catabolic unbalance which leads to muscle atrophy, we found that NeuroHeal sustained a normal activation of protein synthesis after denervation, although with significant differences in protein degradation: downregulation of MuRF1, K48 poly-Ub chains, and p62/SQSTM1. These differences suggested that NeuroHeal promotes a diminution in proteasomal activity and amelioration of the autophagy flux. Moreover, SIRT1 activation by NeuroHeal was necessary to prevent muscle atrophy. These results are in agreement with a recent article describing the protective effect of SIRT1 activity on muscle atrophy [22].

The control of mass in adult skeletal muscle is determined by a dynamic balance between anabolic and catabolic processes triggered by changes in activity or pathological conditions. Muscle hypertrophy is associated with increased protein synthesis induced by activated AKT and mammalian target of rapamycin complex 1 (mTORC1) pathways. Unlike muscle hypertrophy, muscle atrophy always involves a proteostatic shift in favor of catabolic versus anabolic processes. Skeletal muscle protein degradation is executed by several proteolytic systems, including calpains [23], caspases [24], the proteasomal system [25], and the autophagy-lysosomal system [26]. Although, importantly, the selective removal of cellular proteins in muscle largely relies on their ubiquitination followed by proteasomal or autophagy-lysosomal degradation [27,28,29].

The ubiquitin pathway and specific ubiquitin pathway enzymes have been directly implicated in the progression of muscle atrophy. The ubiquitin E3 ligase Muscle-specific RING Finger E3 ligase (MuRF1) is upregulated and increases protein degradation and muscle wasting in numerous muscle atrophy models. MuRF1 knockout mice (MuRF1−/−) is resistant to skeletal muscle atrophy under starvation conditions, post-denervation, and in hindlimb suspension models [30,31], and the use of MuRF1 inhibitors reduces atrophy in cellular models [32]. Although the mechanisms underlying this effect should be analyzed in further detail, we have demonstrated that NeuroHeal downregulates the MuRF1 protein level after denervation, which positioned this agent as a good candidate for clinics.

Autophagy regulates muscle homeostasis, removing protein aggregates and abnormal organelles which otherwise lead to muscle toxicity and dysfunction [4]. Although initially controverted [33,34,35], the use of more specific techniques to analyze the autophagy flux have demonstrated the importance of autophagy to reduce atrophy and its malfunction is associated with muscle wasting. For instance, deficiency in the autophagy-related genes Atg5 or Atg7 is lethal in neonatal mice due to disruption of the supply of transplacental nutrients [4,36]. These findings suggest that autophagy deficiency plays a role in various forms of hereditary muscular dystrophy, including Bessler myopathy, Ullrich congenital muscular dystrophy, and Duchenne muscular dystrophy (DMD) [37]. More recently, Odeh and collaborators demonstrated that autophagy flux was slowed down or even blocked in denervated muscles [38]. Moreover, it has been reported that a correct autophagy flux is fundamental for myofiber survival [39,40] and satellite cells stemness [41]. A key marker to study autophagy flux is the accumulation of both LC3-II and p62, which indicates the blockade of autophagy flux [19]. Our observations are in agreement with the presence of autophagy flux blockade after denervation. Moreover, NeuroHeal, while sustained, increased pULK1 levels and diminished significantly those markers suggesting a deblockade of the flux and thus a promising agent in this area.

Both the ubiquitin-proteasomal system and autophagy degradative pathways are interconnected. Misfolded ubiquitinated proteins are normally degraded via the ubiquitin-proteasomal system, but when this is overloaded or malfunctioning, these proteins are accumulated and are subsequently sequestered into larger structures which become targets for aggrephagy [42,43,44]. The autophagic cargo receptor p62/SQSTM1 is required for the nucleation of such structures [45,46] and is a key factor for both degradative pathways [47,48]. On one side, the accumulation of protein aggregates results in an upregulation of p62, which in turn negatively affects the ubiquitin-proteasomal system, promoting a switch towards aggrephagy. On the other side, high concentrations of K48-linked ubiquitin chains, one of the canonical proteasomal degradation signals [49,50], which can be accumulated upon proteasome deficiency [51], inhibits p62-induced clustering [52]. After denervation, we observed accumulation of both p62 and K48-linked ubiquitin chains, suggesting that both systems are dysfunctional or probably overloaded. With NeuroHeal, both systems are less active or less likely to function correctly probably because the good resolution of autophagy flux may engulf also overloaded proteasomes.

Finally, the effects observed by the addition of NAM were also informative. It has been reported recently that p62 specifically undergoes acetylation, which is required for the formation and subsequent autophagic clearance of p62 clusters [53]. p62 requires acetylation by TIP60 on K420 and K435 to significantly enhance its binding to ubiquitin and p62 clustering during cell starvation. p62 is deacetylated by HDAC6, which is not affected by NAM. However, TIP60 activity is modulated negatively by SIRT1 [54,55]. NeuroHeal increases SIRT1 activity and hence would probably diminish the acetylation activity of TIP60 and consequently slow down p62 clustering. In the presence of NAM, p62 might be overacetylated and might shift toward the avid formation of p62 bodies. Somehow, these observations indicate that softening the autophagy process favors a good resolution. This challenging hypothesis would be very interesting to be tested in the near future. Probably, the controversy of whether to boost autophagy is not the issue to tackle, but its fine-tune activation may yield protective effects. Indeed, we have recently reported that NeuroHeal-fine-tuned autophagy promotes neuroprotection of neonatal motoneurons, improves motor axonal regeneration, and has a role in neuropathic pain [20,56,57,58].

Altogether, our results suggested that NeuroHeal might be an interesting drug to be used in several clinical affectations and leads to muscle atrophy such as neurodegenerative diseases, to peripheral nerve injuries prolonging medical hospitalization, or even to patients with Covid-19 due to the SARS-Cov-2 virus infection.

## 5. Study Limitations and Future Research

The first limitation of this study was to analyze autophagy in a single muscle, in an atrophy model, and in a fixed time window. The flow of autophagy differs widely among tissues, and since it is a dynamic process, it would be interesting to describe its state in different paradigms. However, due to the largely different experimental groups, we had to focus on a muscle at a specific time point after injury. Another limitation of this study was that we did not investigate the involvement of mitochondria in our models, as they are known to play an important role in maintaining muscle homeostasis.

Future studies will determine which cell type is the target of NeuroHeal and will analyze whether NeuroHeal affects the shift in fiber type which occurs during muscle loss. Finally, this work has opened new lines of research to test the therapeutic effect of NeuroHeal on other muscle disorders, such as those triggered by food deprivation.

## 6. Patents

NeuroHeal is currently under patent review.

## Supplementary Materials

The following are available online at https://www.mdpi.com/2073-4409/9/7/1575/s1, Figure S1: NeuroHeal does not affect the diameter of the uninjured myofibers, Figure S2: SIRT1 activity is modulated by NeuroHeal and nicotinamide, Figure S3: Proteasomal subunit 5α is modulated by NeuroHeal in the denervated muscle.
