# Supplementary material for: NeuroHeal Reduces Muscle Atrophy and Modulates Associated Autophagy
Source: Cells. 2020 Jun 28;9(7):1575. doi: 10.3390/cells9071575 (PMC7408527; doi:10.3390/cells9071575)
Supplement: Supplementary file 1 [file cells-09-01575-s001.pdf]

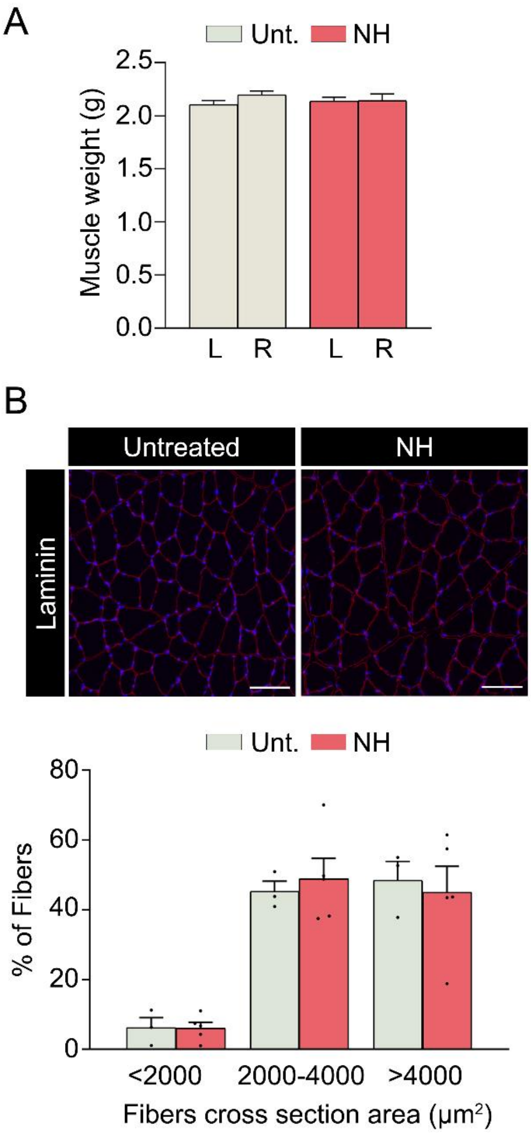

Figure S1. NeuroHeal does not affect the diameter of the uninjured myofibers**.** (**A**) Bar graph of the relative average weight of the ipsilateral and contralateral gastrocnemius (GA) muscle from the different experimental groups: uninjured animals treated for 14 days with vehicle (untreated) or NeuroHeal (n = 4; one-way ANOVA). (**B**) *Up*, representative microphotographs of GA muscle sections stained with laminin (red) and DAPI (blue) from different experimental groups. *Down,* histogram of the cross-sectional area (μm^2) distribution of fibers in GA muscle of different groups (*n* = 4; Kruskal–Wallis, Benjamin, Krieger, and Yekutieti post hoc).

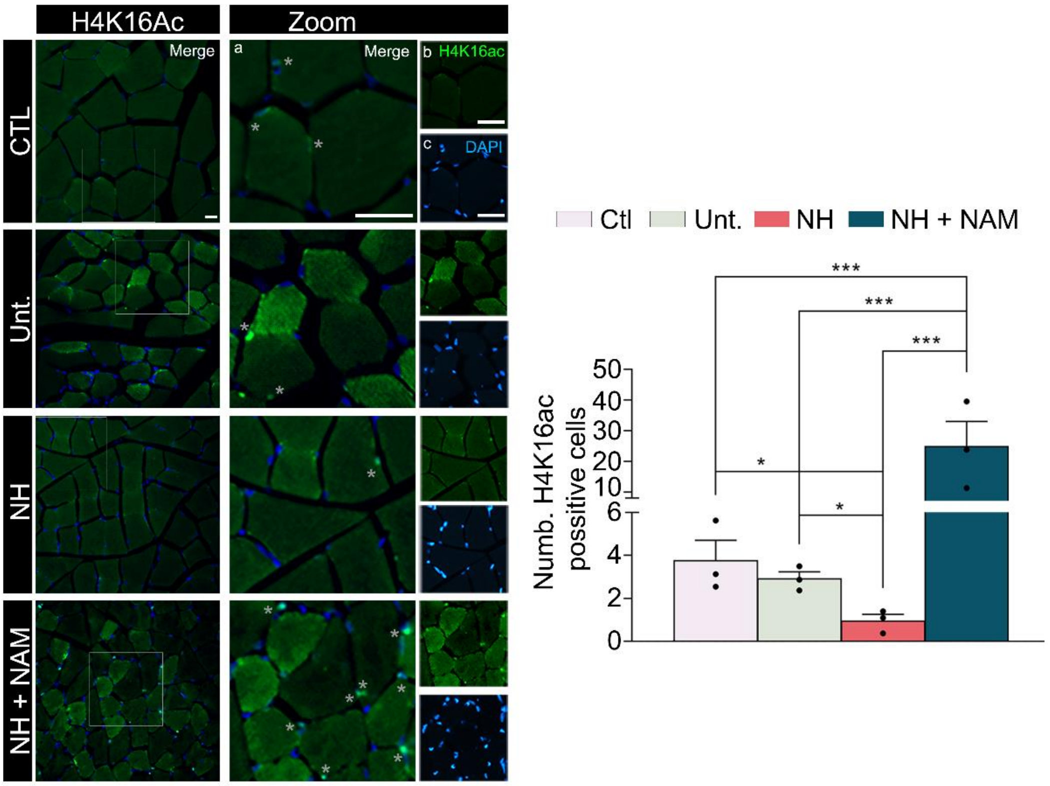

Figure S2. SIRT1 activity is modulated by NeuroHeal and nicotinamide. ***Left***, representative microphotographs of the ipsilateral lesioned gastrocnemius muscle sections revealing the presence of acetylated histone 4 (H4K16Ac, green) and stained with DAPI (blue) from the different experimental groups at 28 dpi: control (CTL), injured untreated (Unt.), injured treated with NeuroHeal (NH), and injured treated with NH plus nicotinamide (NAM). At each condition, panels (**a**–**c**) are zoomed-in images from the squared region of the images of the left. The scale bar is 500 µm and identical for all corresponding microphotographs as represented in the first image panel, the control condition. ***Right***, bar graph of the average number of positive nuclei for H4K16Ac (n = 4 per group, two-way ANOVA, *$p < 0.05$).

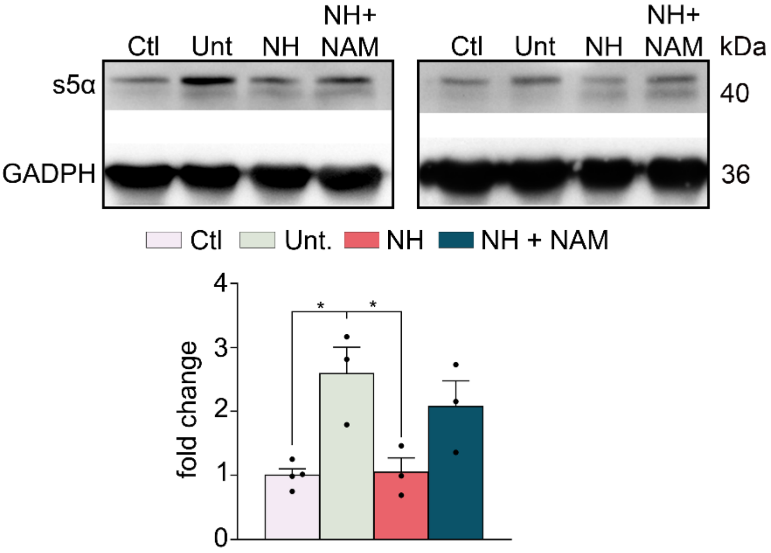

Figure S3. Proteasomal subunit 5*α* is modulated by NeuroHeal in the denervated muscle. Western blots and the associated bar graphs showing the analyses of proteasome subunit 5*α* (s5*α*) protein levels in different experimental groups at 7 dpi (control (CTL), injured untreated (Unt), injured treated

with NeuroHeal (NH), and injured treated with NH plus nicotinamide (NAM)) (n = 3–4; one-way ANOVA, *$p < 0.05$).
